# Corps étranger de l'urètre et dépression mélancolique, les implications diagnostiques aux urgences-à propos d'un cas clinique

**DOI:** 10.4314/pamj.v9i1.71189

**Published:** 2011-06-05

**Authors:** Ali Chouaib, Patrick Cabanis, Thierry Billebaud

**Affiliations:** 1 Service d'urologie, centre hospitalier inter-communal de Creteil, 50 Avenue Verdun 94000, Créteil, France

**Keywords:** Urètre, corps étranger, dépression, urètre, médico-légal

## Abstract

L'auto-insertion de corps étrangers dans l'urètre, est un comportement d'automutilation potentiellement dangereux. Plusieurs cas d'auto-insertion de corps étrangers dans l'urètre, ont été rapportés dans la littérature. Ceci est parfois dû à un trouble mental qui peut être méconnu et doit être systématiquement recherché par l'urologue. Si la notion d'automutilation et/ou les idées suicidaires ne sont pas recherchées dans le milieu hospitalier, et le patient s'automutile ou se suicide par la suite, l'urologue peut être poursuivi en justice pour absence de diagnostic et de traitement, pouvant être interprétés comme erreur médicale.

## Introduction

Les corps étrangers (CE) de l'urètre masculin ont été largement rapportés et discutés dans la littérature. Ceci est souvent du, soit à une curiosité érotique, soit chez des patients présentant des troubles mentaux, ou rarement dans le cadre d'une tentative d’évacuation des urines lors d'une rétention aigue des urines. Le diagnostic est souvent clinique, et l'extraction du corps étranger par voie endoscopique constitue le traitement de référence. En plus du traitement chirurgical, l'urologue a l'obligation d’évaluer l’état psychiatrique du patient, et au moindre doute de le référer au psychiatre pour rechercher une étiologie psychiatrique méconnue dans le milieu du patient, et qui peut être dangereuse [1]. A travers le cas d'un adolescent de 17 ans qui avait une dépression grave et méconnue, découverte suite à l'insertion de six aiguilles à coudre dans son urètre, nous rappelons l'intérêt de l’évaluation psychiatrique de tous les patients se présentant pour insertion de corps étrangers du bas appareil urinaire.

## Patient et observation

Il s'agit d'un adolescent de 17 ans, qui s'est présenté au service des urgences pour une urétrorragie minime et isolée. L'interrogatoire a révélé la notion d'insertion volontaire dans l'urètre de six aiguilles à coudre reliées par un fil, dans un contexte d'ivresse. La famille a confirmé la stabilité mentale de l'adolescent. L'examen clinique a retrouvé le bout du fil extériorisé par le méat urétral. La palpation de l'urètre a permis de localiser les aiguilles, bloquées dans l'urètre, 5 cm au-dessus du méat urétral.

**Figure 1:**
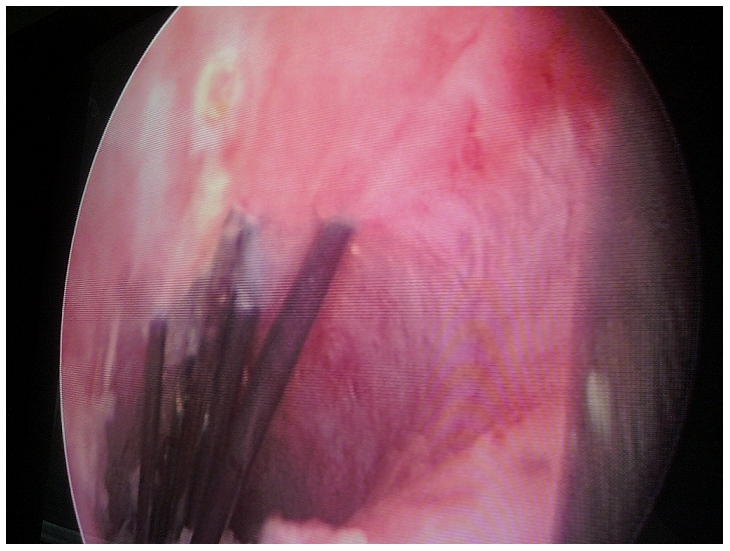
aspect endoscopique de l'urètre avec des aiguilles à coudre incrustées dans la muqueuse urétrale chez un patient de 17 ans reçu au service des urgences pour une urétrorragie minime et isolée

**Figure 2:**
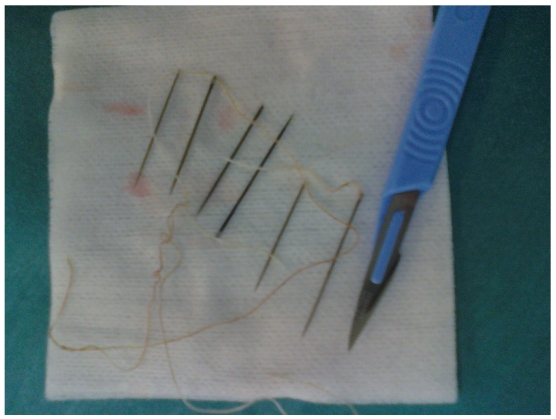
les six aiguilles à coudre et le fil qui les reliait, après extraction endoscopique

Ces aiguilles ont été mises dans le sens inverse, avec le bout pointu dans le sens de la traction, ce qui a rendu impossible leur extraction manuelle par traction sur le fil. Pour compléter l'approche thérapeutique, nous avons réalisé systématiquement une évaluation psychiatrique initiale, pour éliminer une notion d'automutilation d'origine psychiatrique. Le psychiatre a conclu au diagnostic d'une dépression grave avec des idées suicidaires, nécessitant une prise en charge psychiatrique dans un service spécialisé. Le patient fut acheminé au bloc opératoire après vérification de son statut vaccinal et l'administration d'une antibioprophylaxie par voie veineuse. L'urétrocystoscopie et l'utilisation d'une pince à corps étranger a permis de pousser le fil dans la vessie, pour désolidariser les aiguilles et de les retirer une par une, en protégeant le bout pointu entre les maures de la pince. Le fil a été retiré en dernier (Figure 1 et Figure 2).

## Discussion

Plusieurs cas d'insertion de CE dans l'appareil urinaire masculin ont été rapportés dans la littérature, ils sont souvent associés à d'autres comorbidités causales. Les principales étiologies rapportées sont les impulsions érotiques, les troubles psychiatriques surtout dans le cadre d'automutilation, les troubles de la personnalité chez les sujets à personnalité limite, et les intoxications [2]. Malheureusement la majorité des auteurs ne recommandent pas une évaluation psychiatrique systématique.

Alibadi et al [3], a rapporté une série de 18 patients avec une insertion de corps étrangers dans l'urètre, et dont les étiologies étaient les suivantes : l'auto-érotisme chez 6 patients (33%), une cause psychiatrique chez 2 patients (11%), pour soulager une rétention des urines chez 7 patients (39%), et sans cause précise chez 3 patients (17%).

La symptomatologie clinique est polymorphe, variant selon le type et le siège du corps étranger, en effet, elle peut associer un ou plusieurs des signes suivants : dysurie, douleur périnéale, urètrorragie, hématurie microscopique ou macroscopique, rétention aigue des urines [2, 3]. Au niveau de l'urètre antérieur, le CE est souvent accessible à la palpation, permettant ainsi de préciser son siège et ses dimensions.

Si le diagnostic n'est pas clair, il faut demander une radiographie standard pour localiser les CE radio-opaques, et une exploration échographique pour les CE radio-transparents [4]. Le traitement comporte deux volets, d'une part, l'extraction du corps étranger, en utilisant le moyen le moins invasif, tout en évitant les complications, ainsi l'extraction endoscopique doit être toujours tentée en premier.

D'autre part, et vu que les maladies psychiatriques constituent une des causes les plus fréquentes et les plus graves des insertions de CE dans l'urètre, l'urologue a l'obligation de demander systématiquement une évaluation psychiatrique initiale en urgence. Pour ne pas passer à côté d'un trouble psychiatrique grave, nécessitant une prise en charge bien menée. Cette évaluation initiale vise surtout à chercher et à démasquer une dépression méconnue par l'entourage, qui se manifeste d'emblée par des idées d'automutilation et /ou suicidaires [5, 6]. Par conséquence, si ce diagnostic n'a pas été recherché, et le patient s'automutile ou se suicide, l'urologue peut être confronté à des problèmes juridiques liés à l'absence de diagnostic et de traitement, qui peuvent être interprétés comme une erreur médicale [1–5].

## Conclusion

Devant un cas d'insertion de CE au niveau du bas appareil urinaire il faut toujours faire une évaluation psychiatrique visant surtout à rechercher une dépression masquée qui peut être dangereuse. Non diagnostiquée, cette étiologie psychiatrique peut engager la responsabilité juridique de l'urologue.
